# Monitoring the coagulation status of trauma patients with viscoelastic devices

**DOI:** 10.1186/s40560-016-0198-4

**Published:** 2017-01-20

**Authors:** Yuichiro Sakamoto, Hiroyuki Koami, Toru Miike

**Affiliations:** Department of Emergency and Critical Care Medicine, Faculty of Medicine, Saga University, 5-1-1 Nabeshima, Saga City, Saga 849-8501 Japan

**Keywords:** Injury Severity Score, Disseminate Intravascular Coagulation, Abciximab, Tranexamic Acid, Activate Partial Thromboplastin Time

## Abstract

Coagulopathy is a physiological response to massive bleeding that frequently occurs after severe trauma and is an independent predictive factor for mortality. Therefore, it is very important to grasp the coagulation status of patients with severe trauma quickly and accurately in order to establish the therapeutic strategy. Judging from the description in the European guidelines, the importance of viscoelastic devices in understanding the disease condition of patients with traumatic coagulopathy has been widely recognized in Europe. In the USA, the ACS TQIP Massive Transfusion in Trauma Guidelines proposed by the American College of Surgeons in 2013 presented the test results obtained by the viscoelastic devices, TEG® 5000 and ROTEM®, as the standard for transfusion or injection of blood plasma, cryoprecipitate, platelet concentrate, or anti-fibrinolytic agents in the treatment strategy for traumatic coagulopathy and hemorrhagic shock. However, some studies have reported limitations of these viscoelastic devices. A review in the Cochrane Library published in 2015 pointed out the presence of biases in the abovementioned reports in trauma patients and the absence of a quality study in this field thus far. A quality study on the relationship between traumatic coagulopathy and viscoelastic devices is needed.

## Background

Two of the major causes of coagulopathy in trauma patients are coagulopathy secondary to hemorrhagic shock due to massive bleeding and coagulopathy due to severe head injury [1]. The release of tissue factor from the damaged brain tissue is postulated as the cause of coagulopathy due to severe head injury. The fundamental treatment for shock due to bleeding is treatment to achieve hemostasis, but fluid infusion and blood transfusion for long periods of time under insufficient hemostasis may lead to the derangement of hemostasis and the impairment of hemostasis due to hypothermia [2–4]. Therefore, it is important to achieve hemostasis quickly without missing the timing in which the patient is able to cope with physiological changes in the early stage of massive bleeding such as tachycardia, wetness, and coldness in the extremities, and anxiety, rather than cope with hypotension that is a physiological response to massive bleeding. It is also important to perform blood transfusion quickly and appropriately as well as obtain immediate hemostasis for the treatment of hemorrhagic shock that accounts for 90% of incidents of traumatic shock. Since coagulation abnormality which is a physiological response to massive bleeding frequently occurs after severe trauma and is an independent predictive factor for mortality, it is very important to grasp the coagulation status of the patient quickly and accurately in order to establish the therapeutic strategy [1, 5].

It has been recognized that trauma patients are more likely to die from intraoperative metabolic failure than from a failure to complete operative repairs. Damage control surgery (DCS) is surgery that is designed to restore normal physiology prior to normal anatomy in critically ill patients. DCS is important for the treatment of trauma because the development of coagulopathy due to radical hemostasis is fatal [5, 6]. DCS is a therapeutic concept in which hemostasis is achieved in as short a time as possible, physiological function is normalized by postoperative intensive care, and then injury repair is completed by planned reoperation if necessary [7].

For this purpose, the status and degree of coagulopathy must be determined quickly with objective indicators. For example, it is possible that continuation of a surgical operation in a patient with a defect in coagulability fails to save the life of the patient because of uncontrollable bleeding. To avoid such a situation, the criteria known as the trauma triad of death (deadly triad) consisting of hypothermia, metabolic acidosis, and coagulopathy have been proposed for the introduction of DCS [7]. In actual clinical practice, body temperature and acid-base equilibrium can be quickly measured. However, measurement of prothrombin time (PT) that is commonly used as the indicator of coagulability requires more than 60 min before the result is obtained [8]. In addition, it has been said that these indicators reflect the early stage of the coagulation process and that the amount of thrombin produced in this period is only 4% of the total prothrombin [9]. Furthermore, the PT and activated partial thromboplastin time (APTT) do not necessarily reflect the in vivo status of coagulability such as the influence of platelets, because the tests are carried out by adding a blood clotting accelerant to plasma separated from whole blood. The activated clotting time (ACT) that uses whole blood may not reflect the in vivo status of coagulability either, because the test also only reflects the early stage of coagulation similar to PT and APTT [10]. We review the principles of measurement by viscoelastic devices and guidelines for the treatment of traumatic coagulopathy.

## Principle of measurement by viscoelastic devices

### TEG5000 system

The Thrombelastograph (TEG®) is a device that measures the change in viscoelasticity of whole blood without separating out the plasma. The TEG was developed based on a concept reported by Hartert in 1948 [11]. The TEG® was reported as the most rapid available test for providing reliable information on coagulopathy in patients with multiple injuries [12]. Since the usefulness of the TEG® for monitoring coagulability during liver transplantation surgery was reported in 1985 [13], this instrument has been widely used in clinical settings. In addition to the TEG®, the rotational thromboelastometer (ROTEM®) has been used as a common viscoelastic device. A new device has been developed in Japan, and it has a completely different principle of measurement from that of conventional point-of-care (POC) devices to assess coagulation and hemostatic function. This device is the Total Thrombus-Formation Analysis System (T-TAS®) whose measurement principle will be explained elsewhere in this article.

As for the principle of measurement by POC devices, the TEG®5000 and ROTEM® delta optically measure changes in mechanical impedance to a sensor pin generated by clotting-induced change in elasticity of whole blood in a cuvette after the addition of a coagulation accelerant [14, 15].

#### ROTEM system

**Fig. 1:**
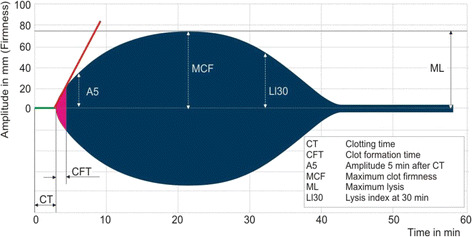
An example of results obtained using the ROTEM system. In the ROTEM® system, the results are displayed in a graph in which the horizontal axis is time (min) and the vertical axis is clot amplitude (mm) based on the firmness of the clot. Various parameters can be measured in real time such as clotting time (CT), clot formation time (CFT), the amplitude at 5 min (A5), maximum clot firmness (MCF), maximum lysis (ML), and lysis index at 30 min (LI30)

**Fig. 2:**
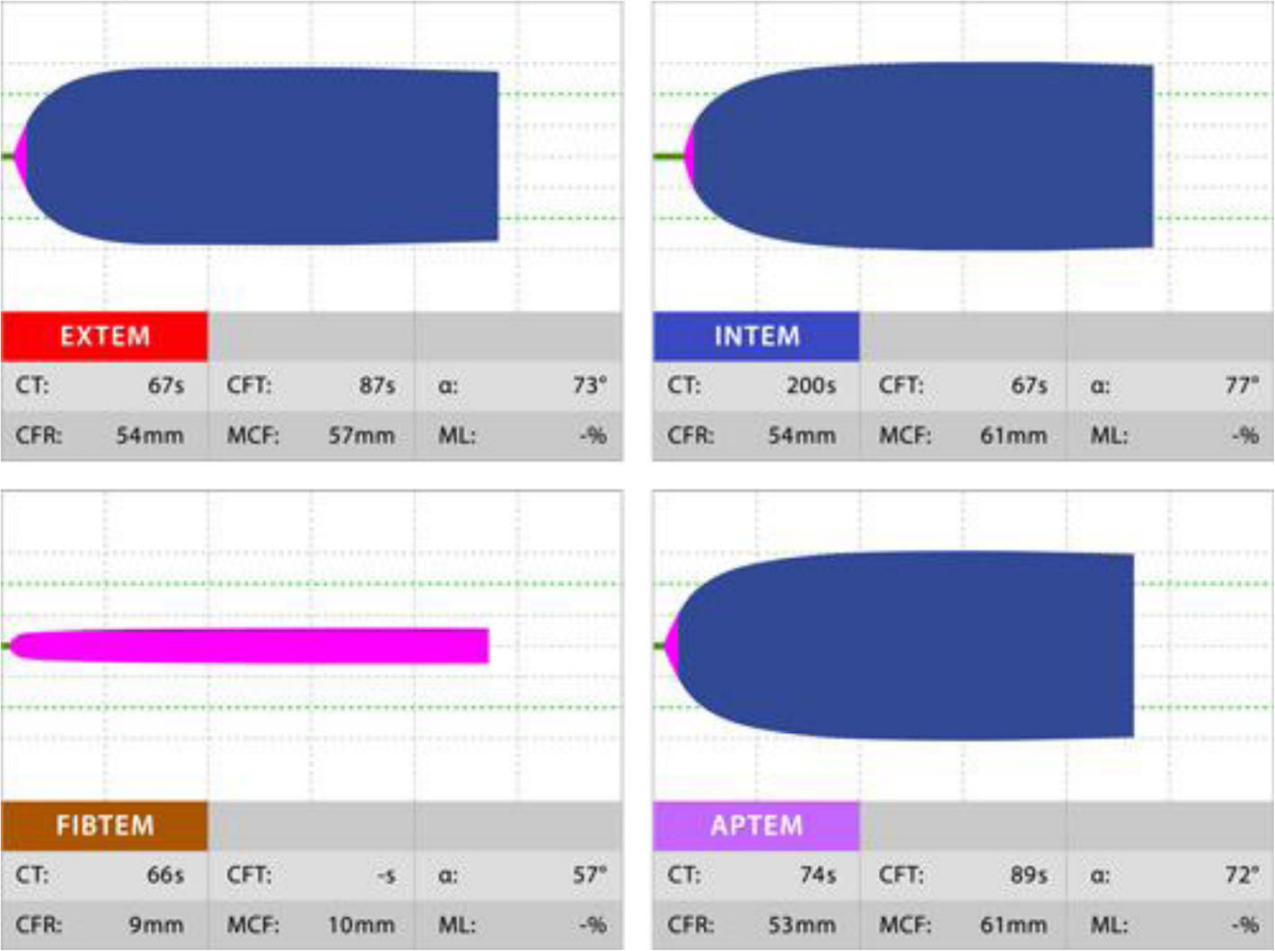
The results in ROTEM in a normal healthy person

**Fig. 3:**
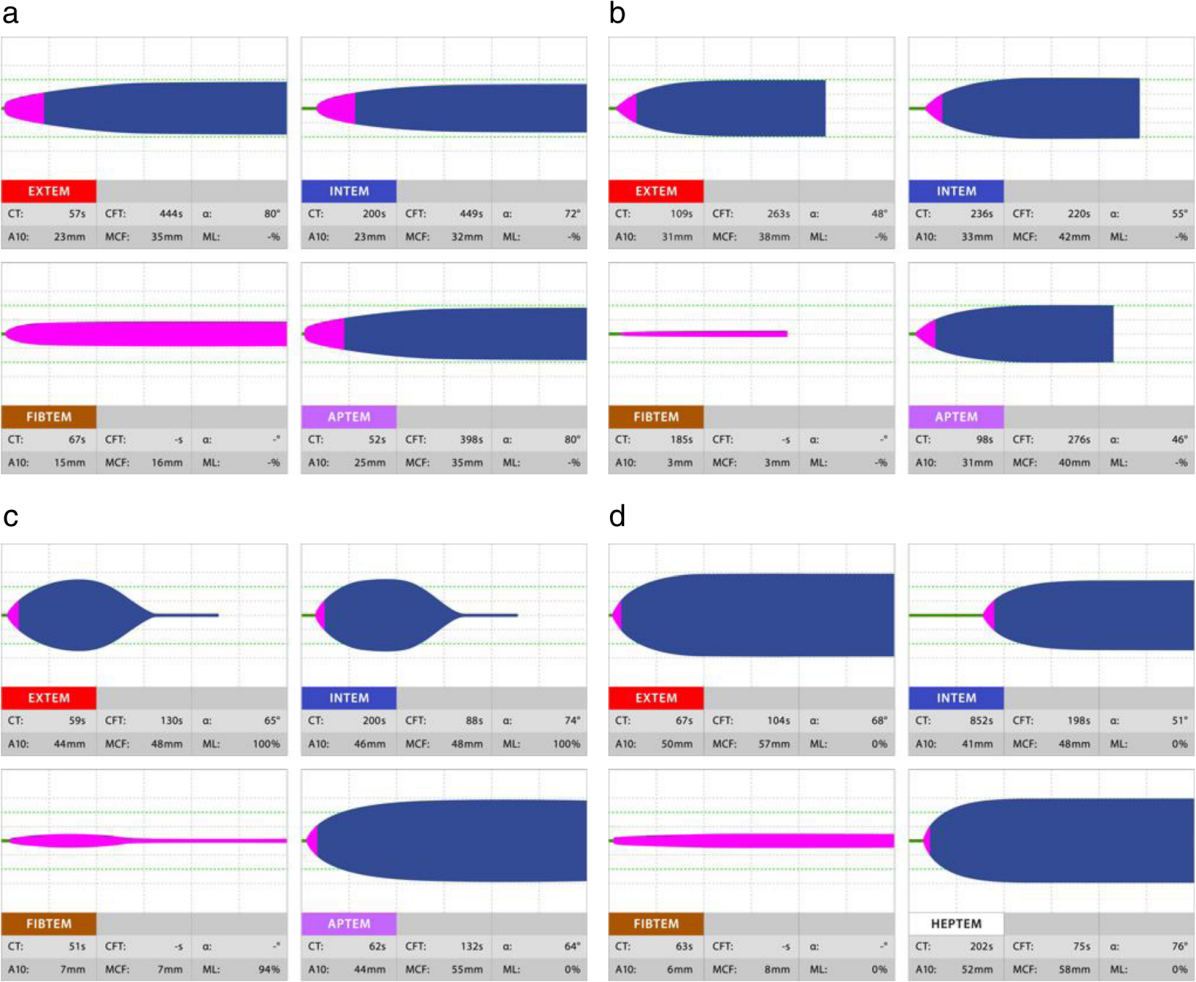
ROTEM results in patients with various hematologic abnormalities. **a** The result of lower clot amplitude in EXTEM indicates platelet deficiency or fibrinogen deficiency or both. The normal result in FIBTEM indicates platelet deficiency. **b** The results of lower clot amplitude in EXTEM and decreased clot amplitude in FIBTEM indicate fibrinogen deficiency. **c** Reduced clot firmness after reaching the MCF indicates the influence of fibrinolysis, and reduced clot firmness by more than 15% from the MCF in EXTEM and FIBTEM but no change in clot firmness after MCF in APTEM indicates hyperfibrinolysis. **d** CT is prolonged in INTEM but does not change or is shorter in HPTEM, and the influence of heparin should be considered

**Fig. 4:**
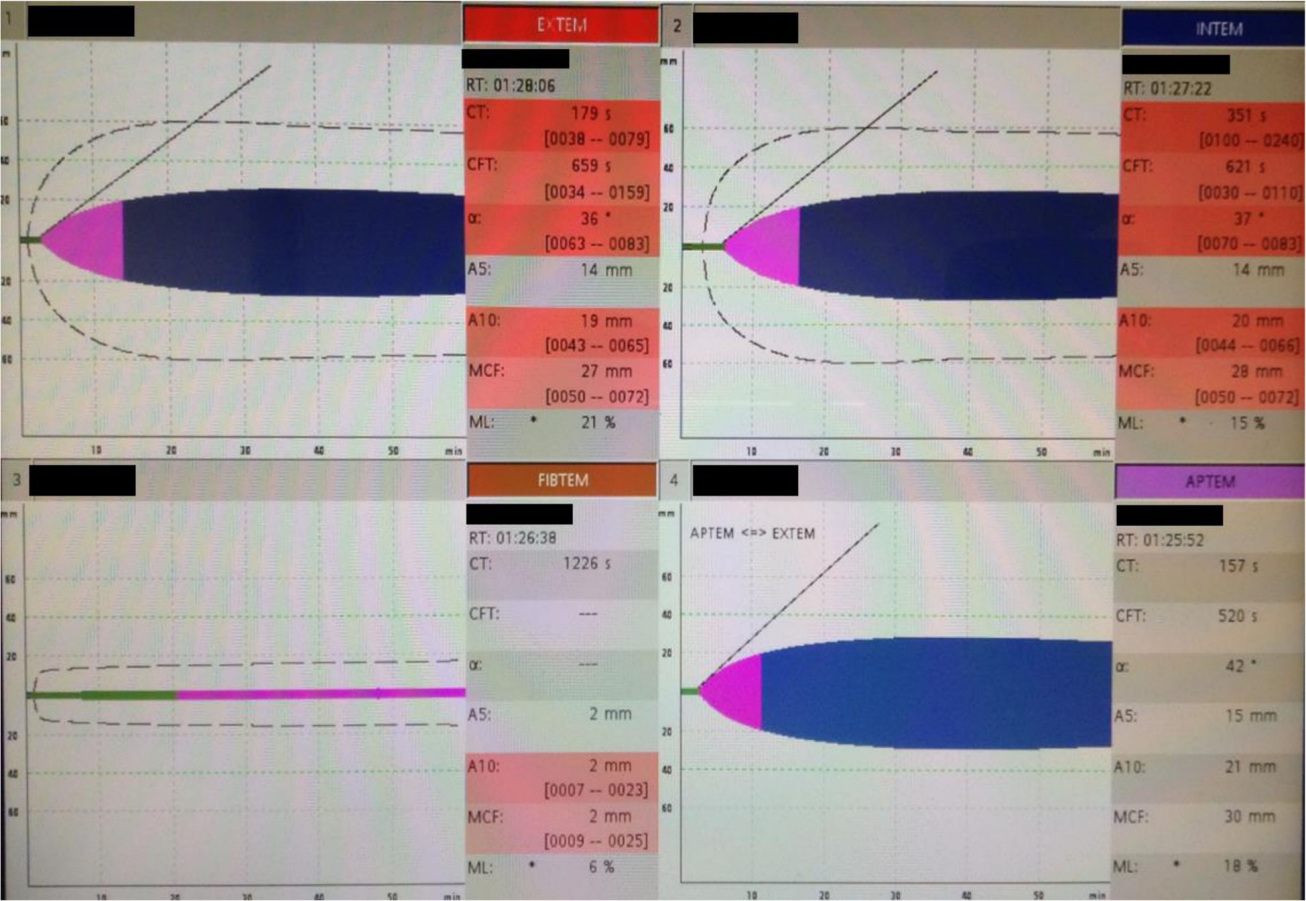
Results using the ROTEM system in a coagulopathic patient with complicated medical conditions. This was a ROTEM result in an 80-year-old woman who complained of vertigo. She had undergone artificial blood vessel replacement surgery for thoracoabdominal aortic aneurysm 8 years previously, and she had chronic hepatitis C, liver cirrhosis (Child-Pugh class B), and chronic atrial fibrillation. The ROTEM test revealed prolonged CT, prolonged CFT, low alpha angle, and low clot amplitude in every test in EXTEM and INTEM. Additionally, significantly reduced clot firmness in FIBTEM indicated fibrinogen dysfunction. This patient was not diagnosed with any acute cerebrovascular disease, and she was discharged on the same day

In the ROTEM® system, the results are displayed in a graph in which the horizontal axis is time (min) and the vertical axis is clot amplitude (mm) which represents the firmness of the clot (Fig. 1). Various parameters can be measured with the ROTEM® system such as the duration from the start of measurement to the beginning of clotting time, duration from the start of clotting to the time when the clot amplitude representing clot firmness reaches 20 mm (clot formation time, CFT) and its angle (α angle), the clot amplitude every 5 min after the beginning of clotting (A 5–30) and its maximum (maximum clot firmness, MCF), the lysis index at 30, 45, and 60 min after the beginning of clotting (LI 30, 45, and 60), and the maximum lysis index (ML) which can be monitored in real time. The results in a normal healthy person are shown in Fig. 2, and the results in representative patients with a clotting abnormality are shown in Fig. 3. In clinical practice, we observe complicated findings in quite a lot of patients with some types of coagulation abnormalities. Case 1 was an 80-year-old woman who complained of vertigo (Fig. 4). She was referred to our hospital because of suspicion of cerebral bleeding. Her past medical history showed that she underwent artificial blood vessel replacement surgery for thoracoabdominal aortic aneurysm 8 years previously, and she had chronic hepatitis C, liver cirrhosis (Child-Pugh class B), and chronic atrial fibrillation. On admission to our emergency department (ED), her consciousness was alert and her vital signs were nearly stable except for slight hypertension. Her blood profiles showed significantly reduced platelet count (3.5 × 10^4^/μL) and fibrinogen level (72.6 mg/dL), prolonged PT-international normalized ratio (INR) (1.47), prolonged aPTT (41.0 s), elevated D dimer level (23.89 μg/mL), and significantly elevated thrombin-antithrombin complex (TAT) level (31.6 ng/mL).We considered that her reduced platelet count also indicated platelet dysfunction. In these data, the parameters of fibrinolysis implied not hyperfibrinolysis but clot retraction because the ML in EXTEM and APTEM was 15% or more [16]. This patient was not diagnosed with any acute cerebrovascular disease, and she was discharged on the same day.

In the TEG®5000 system, tests are carried out by adding premanufactured reagents to a citrated or heparinized whole blood sample in a cuvette. Reagents for TEG®5000 are as follows: kaolin, which is the basic reagent for activating the intrinsic pathway; heparinase that excludes the effect of heparin; tissue factor that activates the extrinsic pathway; batroxobin that induces abnormal fibrin formation; activated factor XIII that promotes fibrin cross-linkage; arachidonic acid (AA) and adenosine diphosphate (ADP) that activate the respective receptor on platelets; and a platelet aggregation inhibitor, abciximab [14]. The TEG®5000 system allows us to conduct six different tests by using different combinations of these reagents. Kaolin TEG is the basic test in TEG® and measures the clotting activity of the intrinsic pathway. Kaolin TEG + heparinase which consists of kaolin and heparinase can detect the influence of heparin. Rapid TEG® that uses kaolin and tissue factor enables the rapid measurement of clot-forming capacity. TEG functional fibrinogen that uses tissue factor and abciximab assesses fibrin-polymerizing activity. Measurement of platelet function is a characteristic function of TEG®, so-called TEG® platelet mapping. The combination of batroxobin, activated factor XIII and AA or the combination of batroxobin, activated factor XIII and ADP can assess the influence of acetylsalicylic acid or a P2Y12 inhibitor, respectively.

**Fig. 5:**
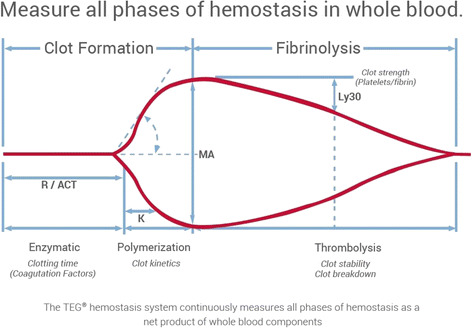
Example of TEG findings. The typical presentation of measurement data obtained by TEG® is shown. The data are displayed in a graph in which the horizontal axis is time (min) and the vertical axis is clot firmness, similar to the ROTEM® system. Parameters are the duration from the start of the measurement to the beginning of clotting (*R*-time), duration from the beginning of clotting to the time when the amplitude of clot firmness reaches 20 mm (*K*-time), clot firmness (MA) and the fibrinolytic index (LY30)

Figure 5 shows the typical presentation of measurement data obtained by TEG®.

The TEG® and ROTEM® systems are based on the same basic principle of measurement. The results that can be obtained from the two systems are summarized in Table 1.

**Table 1:** Comparisons of various parameters between TEG® and ROTEM®

TEG	ROTEM	Interpretation of the finding
*R* (reaction time)	CT (clotting time)	Time to initiation of clot formation
	A (amplitude)	Viscoelasticity of the blood clot. Reflecting the function and counts of platelet and fibrinogen about thrombogenicity of fibrin clot.
*K* (clot kinetics)	CFT (clot formation time)	Time from starting of blood clotting to the clot amplitude reaches at 20 mm. Reflecting the speed of clot polymerization.
*α*	*α* (alpha angle)	Rate of increase of clot amplitude. Reflecting the speed of fibrin clot.
MA (maximum amplitude)	MCF (maximum clot firmness)	Maximum of amplitude. Reflecting the clot strength.
TMA (time to maximum amplitude)		Time to maximum amplitude. Reflecting the clot formation time.
LY	LI (lysis index)	Rate of decrease of clot amplitude. Reflecting the degree of fibrinolytic activity.
CLT (clot lysis time)		Time from maximum to minimum of clot amplitude. Reflecting the degree of fibrinolysis.
	ML (maximum lysis)	Maximum rate of decrease of clot amplitude to MCF.

We introduced the ROTEM® delta in the emergency room of our hospital in January 2013. Clotting time measured in the EXTEM test was a significantly reliable predictor of sepsis-induced disseminated intravascular coagulation (DIC) among 13 sepsis patients [17]. Interestingly, the clotting time measured in EXTEM was strongly correlated with the DIC score of the Japanese Association for Acute Medicine [17]. We assessed the differences in results between traumatized and septic DIC cases that were diagnosed by the same DIC scoring system [18]. This study found that the plasma fibrinogen level and clot firmness measured in the FIBTEM test were significantly different between groups with the same severity. Another paper reported a patient with asymptomatic hyperfibrinolysis diagnosed by ROTEM secondary to anaphylactic shock [19]. In fact, hyperfibrinolysis was significantly associated with elevated serum lactate level (≥4.0 mmol/L) among patients with systemic circulatory insufficiency [20].

#### T-TAS system

T-TAS® is a device that observes the time course of thrombus formation in whole blood flowing in a simulated blood vessel at a constant rate [21]. Since the pressure curve reflects the rate of thrombus formation and thrombus firmness, coagulability and platelet function can be assessed by reading the pressure curve. There are two types of chips having a built-in simulated blood vessel, called PL-chip and AR-chip [22].

**Fig. 6:**
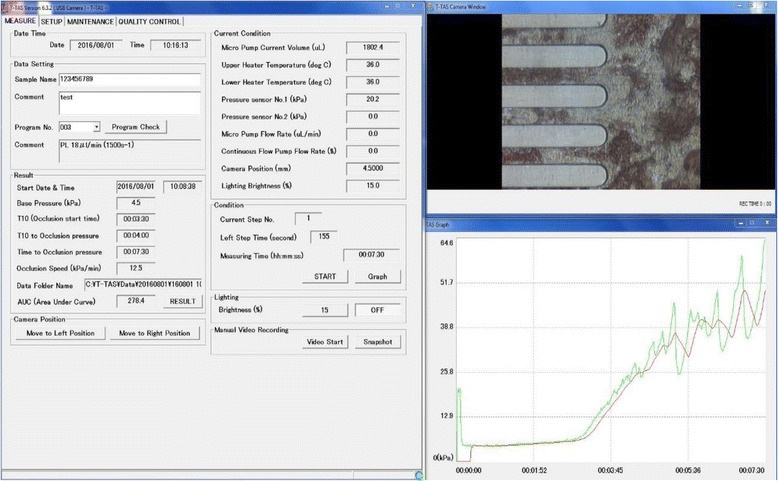
Display screen during measurement with a PL-chip in the T-TAS system. The *left window* shows the measurement conditions such as flow rate of blood and temperature in the simulated vessel. The status of blood flowing can be observed in the *upper right window*. The *lower right window* shows a graph presenting the time course of thrombus formation. Blood flowing in a simulated blood vessel taken by a microcamera can be observed in real time in the *upper right window*. The *lower right window* shows a graph presenting the time course of thrombus formation in which the horizontal axis is time and the vertical axis is the measured pressure. This graph allows us to observe the process of thrombus formation visually. The *left window* shows the measured numerical data and measurement conditions. Measurement conditions are the flow rate of blood flowing in the simulated vessel and the temperature in the vessel, and these flowing conditions can be set freely. Therefore, this device allows us to simulate thrombus formation in various blood vessels in the body. Another chip, the AR-chip, has a built-in simulated blood vessel in which the inner lumen is coated with collagen and tissue factor. After adding Ca^++^ in the simulated vessel, citrated whole blood is activated by the collagen and tissue factor. Then, a very firm thrombus is formed by activated platelets and coagulation factors. Therefore, the AR-chip enables us to assess the cooperative capacity of platelets and the coagulation system in thrombus formation

**Fig. 7:**
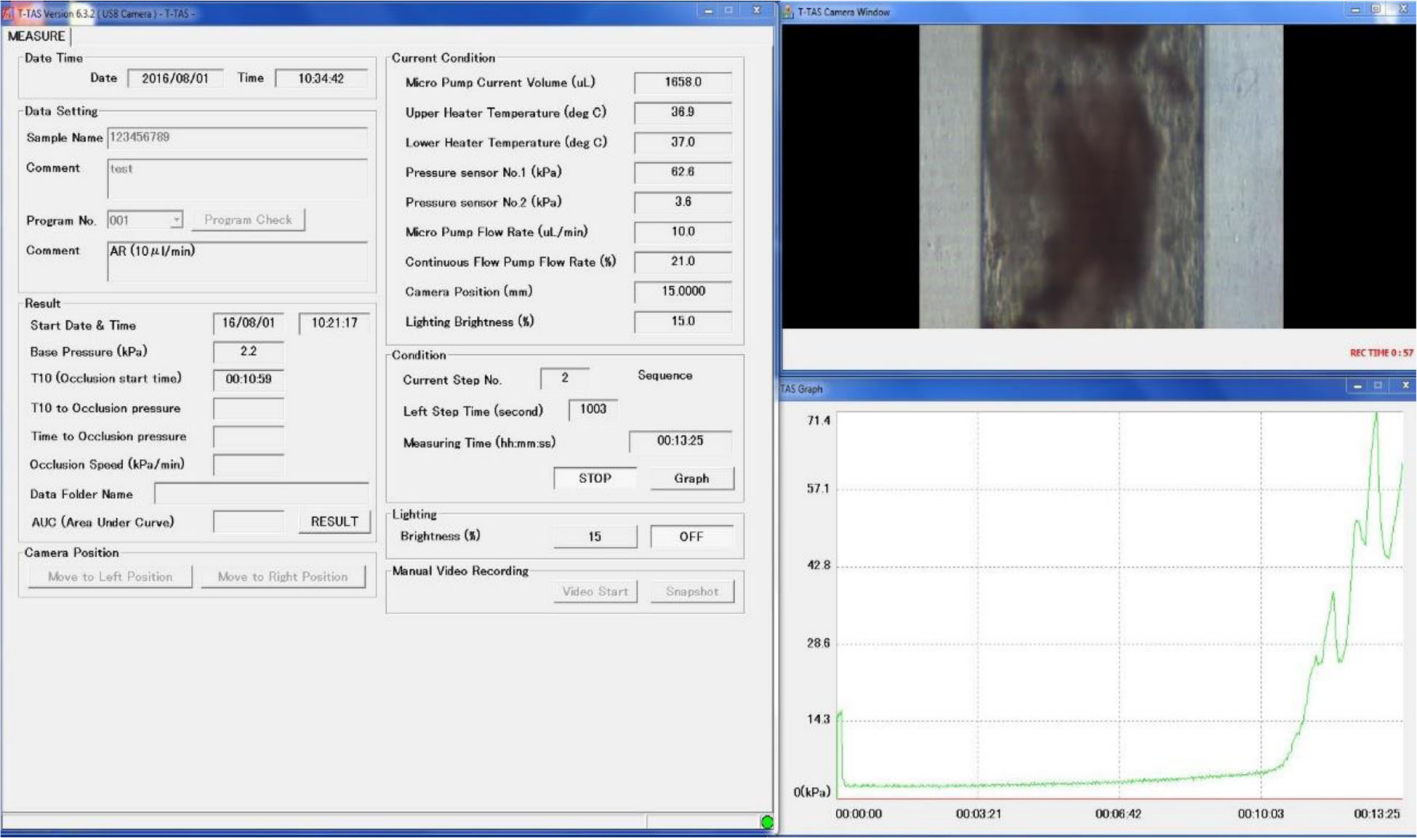
Display screen during measurement with an AR-chip in the T-TAS system. The configuration of the screen is similar to that shown in Fig. 6

**Fig. 8:**
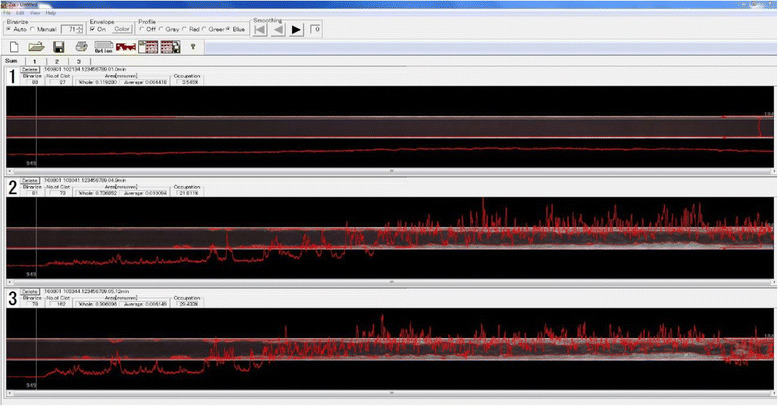
Display screen of T-TAS Zia®. T-TAS Zia® is the built-in software that can analyze thrombus conditions in detail (thrombus formation in the PL-chip can also be analyzed with the software in the most recent model, T-TAS plus®)

The PL-chip which is specialized for the assessment of platelet function consists of a simulated blood vessel in which the inner surface is coated with collagen [23]. Thrombus formation is observed using whole blood anticoagulated with hirudin, a thrombin inhibitor. Platelets bind to collagen on the inner surface of the simulated blood vessel via von Willebrand factor (VWF) to generate shear stress. Platelets activated by shear stress aggregate and trigger thrombus formation in cooperation with fibrinogen and VWF. Figure 6 shows the actual monitor during measurement with a PL-chip. Figure 7 shows the actual monitor during measurement with an AR-chip. The built-in software for analyzing thrombus formation, T-TAS® Zia (Fig. 8), allows us to observe thrombus formation in a simulated vessel of the AR-chip in detail.

In other tests using POC devices and routine coagulation tests in clinical laboratories such as PT and APTT, a coagulation accelerant is directly added and mixed with the whole blood or plasma sample. On the other hand, in the T-TAS® system, collagen or tissue factor that had been coated on the inner surface of a simulated blood vessel activates platelets or the coagulation system in a part of the whole blood sample and then triggers physiological thrombus formation.

**Fig. 9:**
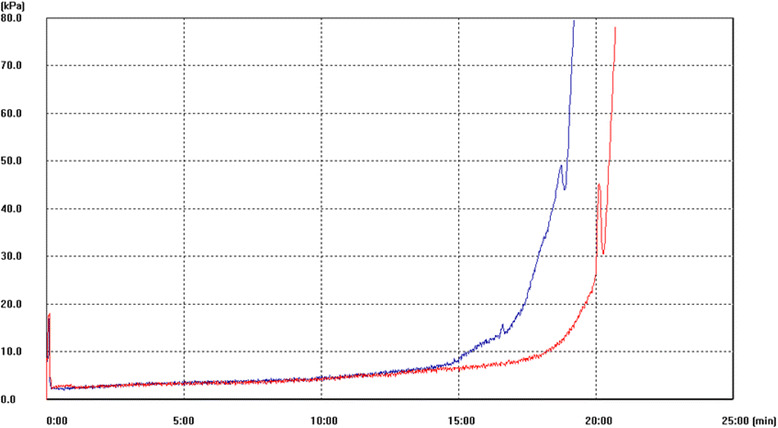
T-TAS® measurement of thrombus formation in a patient who underwent HBOT. The *blue line* represents the result obtained before HBOT, and the *red line* represents the result obtained after HBOT. After HBOT, the coagulation function decreased

We discovered the change in coagulation function of a patient before and after the patient received hyperbaric oxygen therapy (HBOT) [24]. Figure 9 shows a graph of HBOT significantly reduced the clot formation ability of whole blood.

## Viscoelastic devices in the guidelines for the treatment of traumatic coagulopathy in the USA and Europe

The importance of taking into consideration traumatic coagulopathy in the treatment strategy of trauma patients in Europe can be understood from the title of the European guidelines for the treatment of trauma patients. We showed only part of monitoring with viscoelastic devices. Please check other authors’ comments to help the understanding for full guideline. And a European guideline is mentioning as which use is being just recommended, but an American guideline is mentioned until an in-depth numerical analysis. The title of the guidelines published in 2007 [25] was “Management of bleeding following major trauma: the European guidelines,” whereas that published in 2013 [26] was “Management of bleeding and coagulopathy following major trauma: updated European guidelines”; the word “coagulopathy” was added to the title of the more recent guidelines, indicating the growing importance of taking into consideration coagulopathy in the treatment strategy of trauma. The guidelines published in 2013 mentioned that viscoelastic devices were beneficial for establishing the treatment strategy and assessing the status of coagulopathy in patients with hemorrhagic shock (grade 1C). Judging from the description in the European guidelines, the importance of viscoelastic devices in understanding the disease condition of patients with traumatic coagulopathy has been widely recognized in Europe.

In the USA, the ACS TQIP Massive Transfusion in Trauma Guidelines proposed by the American College of Surgeons in 2013 presented the test results obtained by the viscoelastic devices, TEG® 5000 and ROTEM®, as the standard for transfusion or injection of blood plasma, cryoprecipitate, platelet concentrate, or anti-fibrinolytic agents in the treatment strategy for traumatic coagulopathy and hemorrhagic shock [27]. This description indicates that the clinical application of viscoelastic device is more widespread in the USA than in Japan. The guidelines proposed cutoff points using test values obtained by TEG® that indicate the need for transfusion or infusion as follows: plasma replacement if the duration from the start of measurement to the beginning of clotting (*R*-time) > 9 s; administration of plasma or cryoprecipitate (fibrinogen preparation) if the duration from the beginning of clotting to the time when the amplitude of clot firmness reaches 20 mm (*K*-time) > 9 s; administration of cryoprecipitate (or fibrinogen preparation) or plasma if *α* angle <60°; administration of platelet concentrate if the maximum amplitude (MA) < 55 mm; and injection of anti-fibrinolytic agents such as tranexamic acid if the fibrinolytic index (LY30) is >7.5%. The cutoff points using rapid TEG® that indicate the need for transfusion or infusion are as follows: plasma replacement if ACT > 128 s; administration of plasma or cryoprecipitate (fibrinogen preparation) preparations if *K*-time > 2.5 s; administration of cryoprecipitate (or fibrinogen preparation) or plasma if *α* angle <60°; administration of platelet concentrate if MA < 55 mm; and administration of anti-fibrinolytic agents such as tranexamic acid if LY30 > 3%. On the other hand, cutoff points using test values obtained using ROTEM® that indicate the need for transfusion or infusion are as follows: plasma replacement if clotting time >100 s with EXTEM and/or if clotting time >230 s with INTEM; administration of cryoprecipitate (fibrinogen preparation) and/or plasma if MCF < 8 mm with FIBTEM; administration of platelet concentrate if MCF < 45 mm with EXTEM and MCF > 10 mm with FIBTEM; and administration of fibrinolytic agents such as tranexamic acid if ML > 15% with EXTEM.

## Reports on the relationship between the use of viscoelastic devices and the outcome of trauma

Treatment outcome has been considered as an index of the usefulness of information obtained by viscoelastic devices for acute-phase treatment of trauma. There have been a number of reports on the relationship between the test results obtained by viscoelastic devices and outcome in trauma patients [28–31]. One study reported that mortality was 100% in patients manifesting fulminant hyperfibrinolysis with a mean injury severity score (ISS) of 48 [32]. It was also reported that abnormalities of R and MA values measured by TEG® were independent predictive factors for poor outcome [33–36]. It has been demonstrated that prolongation of CFT and a decrease in MCF which indicate a decrease in platelet count measured by ROTEM® were correlated more strongly with poor outcome than with mortality calculated with the Trauma and Injury Severity Score (TRISS) equation [32, 37]. It has been reported that a decrease in fibrinogen level which is detectable in the early stage of coagulopathy was also correlated with poor outcome, suggesting the use of fibrinogen level as the standard for administration of cryoprecipitate and fibrinogen preparations [30]. The study also reported improved survival with infusion and transfusion based on the measurement of the fibrinogen level.

Abnormal findings in platelet mapping analysis with TEG® that represented reduced platelet function were frequently observed among patients who died of head injury [38]. It was also reported that the outcome was better in patients in a hypercoagulable state than in patients in a hypocoagulable state [31].

## Algorithms for trauma care using viscoelastic devices

A specific algorithm for transfusion strategy in trauma patients based on test results obtained with ROTEM® was reported from Parkland Memorial Hospital in 2015, indicating the current spread of viscoelastic devices in clinical practice in the USA [39]. In this algorithm, patients were treated as follows: If ML was prolonged with EXTEM, the patient was judged to have hyperfibrinolysis and tranexamic acid was administered as anti-fibrinolytic treatment. If the clotting time was prolonged with EXTEM, the patient was judged to have reduced coagulability, and a plasma preparation was administered. If the amplitude was reduced with FIBTEM, the patient was judged to have fibrinogen dysfunction and cryoprecipitate or a fibrinogen preparation was administered. If the amplitude was not reduced, the patient was judged to have platelet dysfunction and platelet concentrate was transfused.

On the other hand, Yin et al. [40] reported a goal-directed transfusion protocol based on the results of TEG® in patients with abdominal trauma in Nanjing Hospital, China, in 2014. If the *R* value that represents the time to early clot formation was prolonged, fresh frozen plasma was administered and its dose was decided according to the degree of prolongation. If the *α* angle which is the angle of slope at 20 mm in amplitude and represents the rate of fibrin cross-linkage is depressed, the patient was considered to have fibrinogen dysfunction and cryoprecipitate was additionally administered after fresh frozen plasma infusion. If the *α* angle was normal but MA which represents the strength of the blood clot was reduced, the patient was considered to have platelet dysfunction or a coagulopathy, and platelet concentrate or recombinant factor VII was administered. Several studies conducted in other countries reported the use of viscoelastic devices in trauma care and demonstrated their usefulness for the assessment of traumatic coagulopathy [32, 35, 41–44].

These viscoelastic devices will become an important tool for establishing the treatment strategy in trauma care patients in Japan in the future.

However, some studies have reported limitations of these viscoelastic devices. A review in the Cochrane Library published in 2015 pointed out the presence of biases in the abovementioned reports in trauma patients and the absence of a quality study in this field thus far [45]. The review concluded that PT and INR are the most reliable parameters for monitoring traumatic coagulopathy although these parameters are not perfect. Thus, it mentioned that POC tests should be done with devices used in clinical laboratories because the way of processing was not established for hardly interpretable results obtained with POC devices. At present, the usefulness of viscoelastic devices has been demonstrated only for control of intraoperative bleeding in cardiac surgery, and there has not been favorable evidence for the usefulness of POC devices for transplantation control and improvement of outcomes in trauma patients with other pathologies [46]. To make good use of POC devices in establishing the treatment strategy for patients with traumatic coagulopathy in the future, it is necessary to compare the results obtained from POC devices with the results of PT and INR obtained by laboratory devices. In addition, it may be necessary to clarify and solve the problems of measurement using POC devices and to verify the usefulness of viscoelasticity as a supplementary test item after understanding its characteristics in clinical application.

## Conclusions

Viscoelastic devices will become an important tool in establishing the treatment strategy in trauma care patients in the future. However, some studies have reported limitations of these viscoelastic devices. A quality study on the relationship between traumatic coagulopathy and the results obtained with viscoelastic devices is needed.

## Abbreviations

ACT: Activated clotting time
DCS: Damage control surgery
POC: Point-of-care
PT: Prothrombin time
